# Comprehensive analysis of lncRNA-miRNA-mRNA networks during osteogenic differentiation of bone marrow mesenchymal stem cells

**DOI:** 10.1186/s12864-022-08646-x

**Published:** 2022-06-07

**Authors:** Jialin Liu, Yuan Yao, Jinyong Huang, Hao Sun, Yixuan Pu, Mengting Tian, Meijie Zheng, Huiyu He, Zheng Li

**Affiliations:** 1 Department of Prosthodontics and Implant Dentistry, The First Affiliated Hospital of Xinjiang Medical University, Xin Jiang Uygur Autonomous Region, 830054 Urumqi, China; 2 Affiliated Stomatological Hospital of Xinjiang Medical University, Xin Jiang Uygur Autonomous Region, 830054 Urumqi, China; 3 Stomatology Research Institute of Xinjiang Uygur Autonomous Region, Xin Jiang Uygur Autonomous Region, 830054 Urumqi, China; 4 Department of Trauma Orthopedics, The First Affiliated Hospital of Xinjiang Medical University, Xin Jiang Uygur Autonomous Region, 830054 Urumqi, China

**Keywords:** Bone marrow mesenchymal stem cells, Expression profile, High-throughput RNA sequencing, Long noncoding RNA, Osteogenic differentiation

## Abstract

**Background:**

Long non-coding RNA (lncRNA) plays crucial role in osteogenic differentiation of bone marrow mesenchymal stem cells (BMMSCs), involving in regulation of competing endogenous RNA (ceRNA) mechanisms and conduction of signaling pathways. However, its mechanisms are poorly understood. This study aimed to investigate lncRNAs, miRNAs and mRNAs expression profiles in rat BMMSCs (rBMMSCs) osteogenic differentiation, screen the potential key lncRNA-miRNA-mRNA networks, explore the putative functions and identify the key molecules, as the basis of studying potential mechanism of rBMMSCs osteogenic differentiation driven by lncRNA, providing molecular targets for the management of bone defect.

**Methods:**

High-throughput RNA sequencing (RNA-seq) was used to determine lncRNAs, miRNAs, and mRNAs expression profiles at 14-day rBMMSCs osteogenesis. The pivotal lncRNA-miRNA and miRNA-mRNA networks were predicted from sequencing data and bioinformatic analysis, and the results were exported by Cytoscape 3.9.0 software. Gene Ontology (GO) analysis and Kyoto Encyclopedia of Genes and Genomes (KEGG) analysis were used for functional exploration. Real-time quantitative reverse transcription-polymerase chain reaction (qRT-PCR) was performed to validate lncRNAs, miRNAs and mRNAs.

**Results:**

rBMMSCs were identified, and the osteogenic and adipogenic differentiation ability were detected. A total of 8634 lncRNAs were detected by RNA-seq, and 1524 differential expressed lncRNAs, of which 812 up-regulated and 712 down-regulated in osteo-inductive groups compared with control groups. 30 up-regulated and 61 down-regulated miRNAs, 91 miRNAs were differentially expressed in total. 2453 differentially expressed mRNAs including 1272 up-expressed and 1181 down-expressed were detected. 10 up-regulated lncRNAs were chosen to predict 21 down-regulated miRNAs and 650 up-regulated mRNAs. 49 lncRNA-miRNA and 1515 miRNA–mRNA interactive networks were constructed. GO analysis showed the most important enrichment in cell component and molecular function were “cytoplasm” and “protein binding”, respectively. Biological process related to osteogenic differentiation such as “cell proliferation”, “wound healing”, “cell migration”, “osteoblast differentiation”, “extracellular matrix organization” and “response to hypoxia” were enriched. KEGG analysis showed differentially expressed genes were mainly enriched in “PI3K-Akt signaling pathway”, “Signaling pathway regulating pluripotency of stem cells”, “cGMP-PKG signaling pathway”, “Axon guidance” and “Calcium signaling pathway”. qRT-PCR verified that lncRNA Tug1, lncRNA AABR07011996.1, rno-miR-93-5p, rno-miR-322-5p, Sgk1 and Fzd4 were consistent with the sequencing results, and 4 lncRNA-miRNA-mRNA networks based on validations were constructed, and enrichment pathways were closely related to “PI3K-Akt signaling pathway”, “Signaling pathway regulating pluripotency of stem cells” and “Wnt signaling pathway”.

**Conclusions:**

lncRNAs, miRNAs and mRNAs expression profiles provide clues for future studies on their roles for BMMSCs osteogenic differentiation. Furthermore, lncRNA–miRNA–mRNA networks give more information on potential new mechanisms and targets for management on bone defect.

**Supplementary information:**

The online version contains supplementary material available at 10.1186/s12864-022-08646-x.

## Introduction

The management of maxillofacial non-healing bone defects is always a challenge in the medical field. Over the last decade, tissue engineered bone, made up of seed cells, scaffold materials and cytokines, has brought a treatment light. BMMSCs have been considered as ideal seed cells, and BMMSCs osteogenic differentiation is the key part to bone regeneration. Understanding its molecular mechanisms is significant for therapeutic purposes. Despite researches on BMMSCs osteogenic differentiation are extensive [1], their regulatory mechanisms remain unclear to an extent. Finding and clarifying the key molecules and potential regulatory mechanisms in the differentiation of BMMSCs into osteoblasts is the focus in the field of bone tissue repairment and reconstruction, which could provide crucial information for subsequent studies of clinical targeted therapy on bone defect.

Long non-coding RNA, the length is greater than 200 nucleotides, was considered as “transcription noise” without biological function in the initial stage. However, recent studies investigating lncRNAs showed that they played a cis-regulatory or trans-regulatory role in gene regulation at the pre-transcriptional, transcriptional and post-transcriptional levels, affecting the biological processes of cells and the life activities of the body. Intriguing, emerging evidence indicates that lncRNAs have critical influence on osteogenesis [2, 3], so the specific regulatory mechanism of lncRNAs in osteoblast differentiation process needs to be figured out for the future targeting treatment. Usually, lncRNAs can play as crucial post-transcriptional regulators by binding with microRNAs (miRNAs) to reduce the repression of mRNA targets. Thus, the regulatory relationships among lncRNAs, miRNAs and mRNAs should not be underestimated.

The use of RNA-seq technology combined with bioinformatic analysis has become a scientific mainstream for large-scale screening lncRNAs, miRNAs and mRNAs related to various physiological and pathological processes, providing scientific methodological basis and reliable data support for subsequent research. With no exception, it is also the basis for researches concerning to osteogenic differentiation regulated by various stem cells [4, 5]. Considering that lncRNA is the hotspot of studies in recent years, there are some reports on its expression and functional contributions associated with osteogenesis [6–9], but these are only the tip of the iceberg. Therefore, we need to explore more possible molecules and potential mechanisms related to osteogenesis.

In this study, we detected the lncRNAs, miRNAs and mRNAs expression profiles during 14-day osteogenic differentiation of rBMMSCs using RNA-seq technology. Ten differential upregulation lncRNAs were selected for miRNA response elements (MRE) prediction through sequencing data and bioinformatic analysis. Similarly, the predicted downregulation miRNAs were used to seek for the targeted upregulation mRNAs, and the complicated networks containing the potential lncRNA-miRNA-mRNA targeting relationships were constructed. In addition, we performed GO analysis and KEGG analysis for predicting and understanding the potential mRNAs functions. Based on the predicted networks, we narrowed down the scope and selected 3 lncRNAs, 3 miRNAs and 3 mRNAs for validation. Meanwhile, the probable lncRNA-miRNA-mRNA co-expression networks involving in pathway information derived from verified data were constructed. Our findings may provide a theoretical foundation for future studies on modulation of lncRNAs in BMMSCs osteogenic differentiation, making preliminary preparations for the application of lncRNAs to target therapy on bone regeneration.

## Materials and methods

### Cell isolation and culture of rBMMSCs

 All experiments were approved by the Animal Research Committee of Xinjiang Medical University (approval no. IACUC20170706-04). A total of 6 male SD rats (4 weeks old), weighing about 100g, were provided by the Xinjiang Medical University Animal Experimental Center. The rBMMSCs were isolated from 6 rats using the whole bone marrow adherence method. The cells were cultured in α-MEM medium (Gibco, USA) supplemented with 10% FBS (Gibco, USA), 1% antibiotics (Biological Industries, Israel) and 1% L-Glutamine solution (Biological Industries, Israel) at 37°C cell culture incubator (Thermo, USA) with humidified atmosphere containing 5% CO_2_. Culture medium was replaced every two days, and cells were passaged at a 1:2 ratio after reaching 80%~90% confluence. The cells (5 × 10^5^ cells/25 cm^2^ culture flask) were transferred to next passage using 0.25% trypsin (HyClone, USA) for up to five passages. Usually, the third passage cells were uniformly used for subsequent experiments.

### Flow cytometry analysis

The third passage cells were collected to identify the phenotypes. When rBMMSCs washed with PBS twice and filtered to resuspend in 100µl PBS, four kinds of primary antibodies including PE anti-rat CD44 (Abcam, ab23396, USA), PE anti-rat CD29 (Biosciences, 562154, USA), PE anti-rat CD45 (Biolegend, 202207, USA) and FITC anti-rat CD34 (SantaCruz, sc-7324, USA) were added in and incubated at 4°C for 30min without light. The antigen expressions can be analyzed by flow cytometer (BD FACSCalibur, USA) according to the manufacturers’ instructions.

### Multilineage differentiation induction

The isolated cells were cultured in 10%α-MEM medium for the third generation with about 70%~80% confluency, and then replaced with osteo-inductive medium or adipo-inductive medium for the multilineage differentiation.

The osteo-inductive medium (Cyagen, USA) containing 175ml SD rat bone marrow mesenchymal stem cell osteogenic differentiation basal medium, 20ml FBS, 2ml β-glycerophosphate, 400µl L-ascorbic acid, 2ml glutamine, 2ml penicillin-streptomycin solution and 20µl dexamethasone, was used for cells incubation with 5% CO_2_ humidified atmosphere at 37°C. Cultures medium received change every three days.

The adipo-inductive medium (Cyagen, USA) containing SD rat bone marrow mesenchymal stem cell adipogenic differentiation medium A (175ml SD rat bone marrow mesenchymal stem cell adipogenic differentiation basal medium, 20ml FBS, 2ml glutamine, 2ml penicillin-streptomycin solution, 400µl insulin, 200µl isobutyl-methylxanthine, 200µl rosiglitazone, and 200µl dexamethasone) and SD rat bone marrow mesenchymal stem cell adipogenic differentiation medium B (175ml SD rat bone marrow mesenchymal stem cell adipogenic differentiation basal medium, 20ml FBS, 2ml glutamine, 2ml penicillin-streptomycin solution, and 400µl insulin), which were used alternately in three to five cycles until fat droplets formation.

### Multiple staining procedure

To avoid floating the cells during osteogenic or adipogenic induction, the culture dish surfaces were coated with 0.1% gelatin solution (Cyagen, USA) before seeding cells. After 14-day osteogenic induction, the cells were stained with Alizarin red and Von Kossa staining, investigating its osteogenic differentiation potential. The staining results were observed and recorded under a light microscope (Leica, Germany) at a magnification of 50×. Meanwhile, after 21-day adipogenic induction, the cells were stained with Oil Red O staining for its adipogenic differentiation potential. The staining results were observed and recorded at both 50× and 200× magnifications.

A solution of 0.1% Alizarin red S (Cyagen, USA) at PH=4.2 was used to stain the calcified nodules. Firstly, the samples were washed twice with PBS and fixed with 4% paraformaldehyde for 30min. Then, they were washed twice with PBS and stained Alizarin red solution for 5min. After rinsing the excess dye twice with PBS, the red calcified nodules can be seen.

The Von Kossa staining kit (Solarbio, China) was exerted to stain the calcified nodules. Similarly, the samples were washed with PBS and fixed with 4% paraformaldehyde for 30min. After washing, they were treated with Von Kossa silver solution and irradiated with ultraviolet light for 10min. Next, they were washed with distilled water for 1min and dyed with hypo solution for 2min. The brown or black calcified nodules can be manifested after washing the excess dye.

The Oil red O stock solution (Cyagen, USA) was diluted with deionized water (3:2), and the supernatant was used to stain fat droplets after 250×g centrifuging. The cells were washed with PBS, and fixed with 4% paraformaldehyde for 30min. Deionized water was used to wash the fixed solution. Then, cells were stained with Oil red O filtered solution for 30min and rinsed twice with PBS. The fat droplets can be stained in red.

### Sample preparation

rBMMSCs on passage three were seeded at a density of 3 × 10^5^ cells in 25 cm^2^ culture flask with 10% complete medium. On one hand, when the cells reached up to 70%~80% confluency, the osteo-inductive medium was used for osteogenic-specific induction group. On the other hand, rBMMSCs under the same conditions were cultured in 10% complete medium to serve as the corresponding control group. Three osteogenic-specific induction groups and three control groups were included totally for 14-day incubation.

### RNA extraction and quality examination

RNA extraction was applied with Trizol (Sigma-Aldrich, USA) traditional procedure. RNA degradation and contamination was detected by 1% agarose gels. RNA purity was checked using the kaiaoK5500® Spectrophotometer (Kaiao, Beijing, China). RNA concentration was tested by Qubit®3.0 Flurometer (Life Technologies, CA, USA). RNA integrity was assessed using the RNA Nano 6000 Assay Kit on Bioanalyzer 2100 system (Agilent Technologies, USA).

### Preparation and examination of the cDNA library for high-throughput RNA sequencing

A total amount of 3µg RNA per sample was used as initial material for sample preparations to construct lncRNA, miRNA and mRNA libraries. Ribosomal RNA was removed using Epicentre Ribo-Zero™ Gold Kits (Epicentre, USA). The sequencing libraries were generated following manufacturer recommendations with varied index label by NEBNext® Ultra™ Directional RNA Library Prep Kit for Illumina (NEB, Ispawich, USA). RNA concentration of library was preliminary measured using Qubit® 3.0, and then dilute to 1ng/µl. Insert size was assessed using the Agilent Bioanalyzer 2100 system (Agilent Technologies, USA), and qualified insert size was accurate quantified using Taqman fluorescence probe of AB Step One Plus Real-Time PCR system (Library valid concentration > 10nM). After cluster generation, the sequencing of the cDNA library was carried out by OE Biotech (Shanghai, China) on an Illumina Hiseq 4000 platform and 150bp paired-end reads were generated.

### Quality control of transcriptome sequencing

Low-quality tags in the raw data were removed, and ribosomal RNA data were also removed from the remaining data by alignment. The 150bp paired-end reads were checked with FastQC. For transcriptome project, an overall assessment was carried out through RSeqQC from three aspects: sequencing saturation, randomness of sequencing library construction, and enrichment of reads in different elements of the genome.

### Identification of lncRNAs

StringTie software was used to reconstruct transcripts in each sample based on probability model.

Cuffcompare software was used to compare merged transcripts with reference transcripts. The known-lncRNAs and similar transcripts to other ncRNAs and mRNAs were screened out, retaining transcripts on the basis of lncRNAs characteristics. The length > 200bp and exon number ≥ 2 were adopted to obtain candidate lncRNAs. Moreover, CPC (Coding Potential Calculator) analysis [10], CNCI(Coding-Non-Coding Index) analysis [11], Pfam protein domain analysis [12] and PLEK analysis [13] were applied for predicting the coding ability of candidate lncRNAs comprehensively. Corresponding results were displayed through Venn diagrams.

### Screening on differentially expressed lncRNAs, miRNAs, and mRNAs

Gene expression was calculated using FPKM method [14], which considered the impact of sequencing depth and gene length on fragment count, can be directly used for comparing gene expression between different samples. The differential expression of genes was calculated using the negative binomial distribution test in DESeq software [15]. The difference significance test was conducted and the gene expression was displayed by basemean value. The osteogenic-specific induction groups and control groups were compared to search for differentially expressed genes, including lncRNAs, miRNAs and mRNAs, using |Foldchange|≥2 and *P* < 0.05 as the limitations.

### Interactive networks prediction based on sequencing data and bioinformatic analysis

Based on differentially expressed lncRNAs sequencing results, 10 significantly up-regulated lncRNAs were selected for predicting targeted down-regulated miRNAs in sequencing data. The lncRNA-miRNA interactions were predicted through the *P* value information of sequence binding sites calculated from RNA22 v2 microRNA target detection [16] database, and the top 5 miRNAs were chosen for each lncRNA to construct lncRNA-miRNA networks. Additionally, the up-regulated mRNAs in sequencing data which could be targeted to the predicted miRNAs were obtained by intersecting with the predicted merged data derived from Targetscan 8.0 [17, 18] and miRDB [19, 20] databases. The intersected data were exported by Venn analysis, and miRNA-mRNA networks were constructed using Cytoscape3.9.0 software.

### GO enrichment and pathway enrichment

GO enrichment database [21, 22] which provides three categories on biological process (BP), cellular component (CC), and molecular function (MF) was performed to describe meaningful annotation of genes and gene products attributes, and *P* < 0.05 was considered statistically significant. The top 20 GO terms for each category ranked by − log_10_ (*P* value), which pattern can reflect gene count and enrichment score.

Pathway enrichment was performed by the latest Kyoto Encyclopedia of Genes and Genomes database [23, 24], searching for probable cellular pathways that may be associated with the differentially expressed genes, and *P* < 0.05 was considered statistically significant. The top 30 KEGG enrichments ranked by − log_10_ (*P* value) showed the significance order of the correlated biological pathway.

### Validation of lncRNAs, miRNAs and mRNAs

3 lncRNAs, 3 miRNAs and 3 mRNAs were candidate for validation. Total RNA was extracted from rBMMSCs that either 14-day osteogenic-specific induction groups or control groups using miRNeasy Mini Kit (Qiagen 217004, Germany). The concentration and purity of RNA were measured by NanoDrop 1000 Nucleic Acid Protein Analyzer (Thermo, USA). Concentrations were greater than 500ng/µl. Additionally, A260/280 = 1.8 ~ 2.0 and A260/230 = 2.0 ~ 2.2 were detected. RevertAid First Stand cDNA Synthesis Kit (Thermo K1622, USA) and miRcute Plus miRNA First-Strand cDNA Kit (TIANGEN KR211, Beijing, China) were used for synthesizing cDNA.

The qRT-PCR was performed in a 20µl reaction volume with QuantiNova SYBR Green PCR Kit (Qiagen 208054, Germany) using ABI QuantStudio 6 Flex system (Thermo Fisher, USA). According to kit instruction, it included 10µl SYBR Premix Ex Taq TM, 10µM forward primer, 10µM reverse primer, 1µg template cDNA, and the rest of the DNase/RNase-free water. Real-time quantitative reverse transcription polymerase chain reaction (qRT-PCR) was initiated at 95°C for 2min and then carried out for total 40 cycles (95°C for 5s, 60°C for 30s). Similarly, for miRNAs validation, corresponding supplements were mixed according to miRcute Plus miRNA SYBR Green qPCR Kit (TIANGEN FP411, Beijing, China) instruction. The procedure was acted on 95°C for 15min, and then set on 94°C for 20s and 60°C for 34s for total 40 cycles. GAPDH was considered as the internal control for lncRNAs and mRNAs, while those of miRNAs were normalized with U6. To clarify the expression stability of normalization gene between control and osteogenic differentiation, we calculated the average Ct values ± standard deviation of the internal control U6 as 15.79 ± 0.46 and 14.25 ± 0.40, respectively. The relative expression of each lncRNAs, miRNAs and mRNAs were calculated by the 2^−△△Ct^ method [25]. Results were harvested from three independent repeated experiments. The primers listed in Table 1 were purchased from OE Biotech (Shanghai, China). Based on the verified data, corresponding lncRNA-miRNA-mRNA networks were constructed with pathways information using Cytoscape 3.9.0 software.

**Table 1:** The primers of lncRNAs, miRNAs and mRNAs for qRT-PCR validation

Gene name	Primer (5’ to 3’)	Primer Sequence
Tug1(rat)	Forward	TGCTGAAGTTGTTTGCCTGC
	Reverse	TCCTTGGTGGAATTGGGCAC
AABR07011996.1(rat)	Forward	CGCTCACATTGCTCCTTGTT
	Reverse	CAGGCTCTGCACAACTGTTT
AABR07060133.1(rat)	Forward	GCTAATGGGAGTCTTGCAGC
	Reverse	AAGTAGTTCTCTGCGGCCTT
Nedd4l(rat)	Forward	CGCCGTGCTGTGAAAGATACCC
	Reverse	GTGTGACTTTGTGTTGTGGTTTGGG
Sgk1(rat)	Forward	AAGGAAACGTCAGTGCTCG
	Reverse	GGAGTTGTTGGCAAGCTTCTG
Fzd4(rat)	Forward	GTGGATCCTCGTGCCTTTCA
	Reverse	GGCATGGATAGCAGGGGTTT
GAPDH(rat)	Forward	GGCACAGTCAAGGCTGAGAATG
	Reverse	ATGGTGGTGAAGACGCCAGTA
ALP(rat)	Forward	AACGTGGCCAAGAACATCATCA
	Reverse	TGTCCATCTCCAGCCGTGTC
Runx2(rat)	Forward	CCATAACGGTCTTCACAAATCCTT
	Reverse	CTGTCTGTGCCTTCTTGGTTC
OCN(rat)	Forward	GGACCCTCTCTCTGCTCACTCTG
	Reverse	ACCTTACTGCCCTCCTGCTTGG
rno-miR-129-5p(rat)	Forward	CAGCTTTTTGCGGTCTGGGCTTGC
rno-miR-93-5p(rat)	Forward	GCGCAAAGTGCTGTTCGTGCAGGTAG
rno-miR-322-5p(rat)	Forward	CGCGTGCGCAGCAGCAATTCATGTT

### Statistical analysis

All data were representative of experiments with similar results performed in triplicate. Mean ± SD and independent t test was applied for values expression. Data were analyzed using GraphPad Prism 8.0 software with *P* < 0.05 being regarded as statistically significant.

## Results

### rBMMSCs identification and 14-day osteogenic induction

**Fig. 1:**
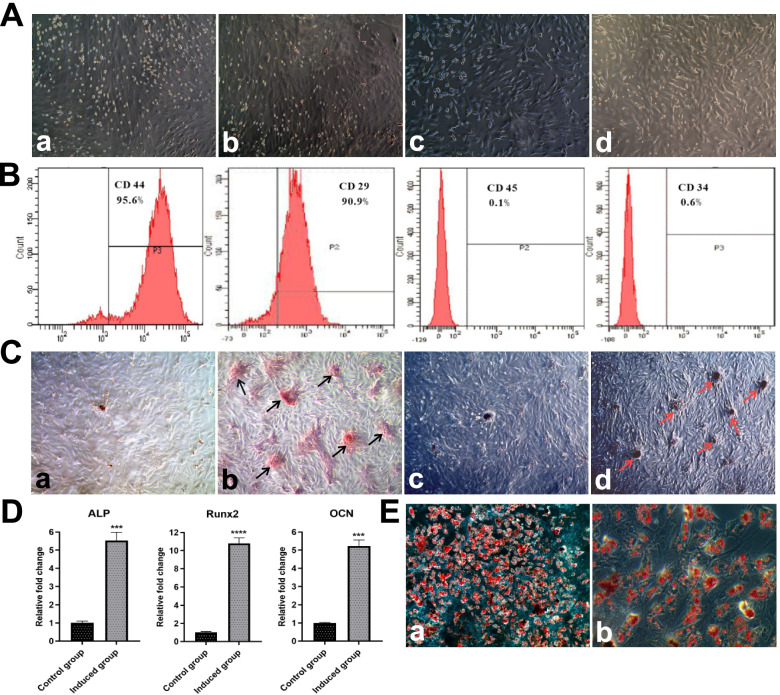
Culturation and identification of rBMMSCs. **A**, The culturation of rBMMSCs. **a**, Passage 0; **b**, Passage 1; **c**, Passage 2; **d**, Passage 3. **B**, Identification of rBMMSCs by four cell surface markers. **C**, Staining identification on 14-day osteogenic differentiation of rBMMSCs. **a**, Alizarin red staining in control group; **b**, Alizarin red staining in osteo-inductive group; **c**, Von Kossa staining in control group; **d**, Von Kossa staining in osteo-inductive group. **D**, qRT-PCR validation on 14-day osteogenic differentiation of rBMMSCs. **E**, Oil Red O staining identification for 21-day adipogenic differentiation of rBMMSCs. **a**, A magnification of 50×; **b**, A magnification of 200×. Independent t test was used in qRT-PCR validation. *P* < 0.05 considered significant difference, and ns indicated that the difference is not statistically significant

BMMSCs were isolated from SD rat femur and tibia bone marrow tissues. The cells at passage three were identified by cell surface markers and multipotentiality (Fig. 1A). Flow cytometry analysis revealed that rBMMSCs positively expressed CD44 (95.6%), CD29 (90.9%), and negatively expressed CD45 (0.1%), CD34 (0.6%) (Fig. 1B). Compared with cells cultured in 10%α-MEM medium, cells cultured in osteo-induced medium for 14 days showed osteogenic differentiation potential owing to the increased calcified nodules stained by Alizarin red and Von Kossa staining kits (Fig. 1C). Also, the osteogenesis related mRNAs expression, including alkaline phosphatase (ALP), runx-related transcription factor 2 (Runx2), and osteocalcin (OCN) increased significantly in 14-day osteo-induced groups when compared with control groups (Fig. 1D). Furthermore, cells cultured in adipo-induced medium for 21 days showed adipogenic differentiation ability by Oil red O staining (Fig. 1E). All these results indicated that rBMMSCs with multidirectional differentiation potential were successfully obtained. The 14-day osteo-induced rBMMSCs were undergoing osteogenic differentiation, providing a firm foundation for the sample preparation of RNA-seq.

### Detection on lncRNAs

**Fig. 2:**
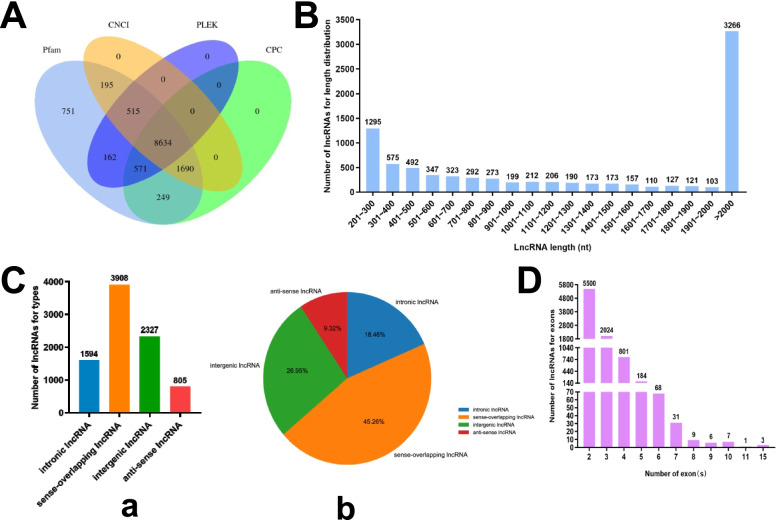
Detection on lncRNAs characteristics. **A**, Identification of lncRNAs based on four algorithms. **B**, LncRNAs length distribution. **C**, Classification of lncRNAs based on their genomic location. **a**, Number of lncRNAs for each type. **b**, Proportion of lncRNAs for each type. **D**, Number of lncRNAs for exons

cDNA libraries were constructed for RNA-seq to profile 14-day osteogenesis correlated lncRNAs, miRNAs and mRNAs information. In total, 8634 lncRNAs were detected based on four algorithms, such as CPC, CNCI, Pfam, and PLEK analysis, the results were exported by Veen analysis (Fig. 2A). According to their length distribution, the majority lncRNAs were shorter than 2000nt, and its number around 1200nt were roughly equal (Fig. 2B). Based on the lncRNAs genomic location, 1594 intronic (18.46%), 3908 sense-overlapping (45.26%), 2327 intergenic (26.95%) and 805 anti-sense (9.32%) lncRNAs were classified (Fig. 2C). The type of sense-overlapping lncRNAs accounted for the largest proportion, while the proportion of anti-sense lncRNAs was the lowest. In terms of the number of lncRNA exons, the number of lncRNAs with 2 exons accounted for the most, followed by 3 and 4 exons. (Fig. 2D).

### lncRNAs, miRNAs and mRNAs expression profiles

**Fig. 3:**
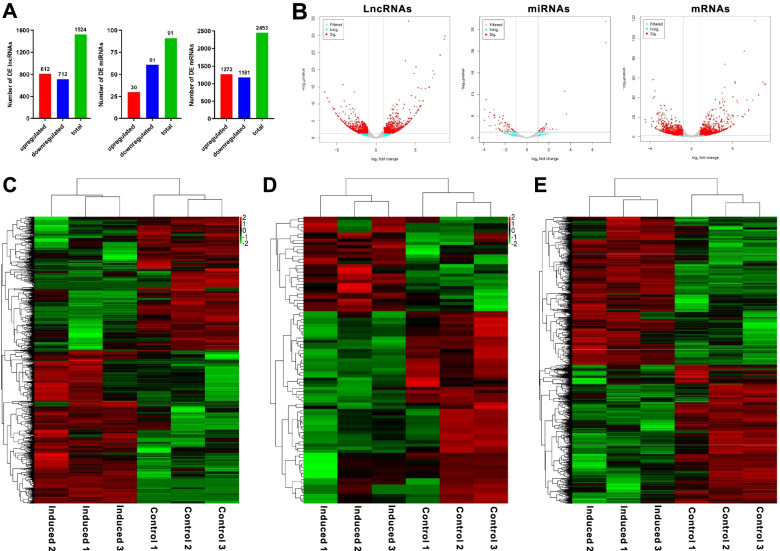
Expression profiles of lncRNAs, miRNAs and mRNAs in 14-day osteogenic differentiation of rBMMSCs. **A**, Detection of differentially expressed lncRNAs, miRNAs and mRNAs. **B**, Three volcano maps of differentially expressed lncRNAs, miRNAs and mRNAs with the standards of *P* < 0.05 and |Fold change|>2. Filtered transcripts were in gray, transcripts with non-significant differences were in blue, and transcripts with significant differences were in red. log_2_ Fold change was displayed on the horizontal axis, and -log_10_ (*P* value) was displayed on the vertical axis. **C**, lncRNAs expression profile in heat map. **D**, miRNAs expression profile in heat map. **E**, mRNAs expression profile in heat map. These three heat maps were constructed based on expression values of all expressed lncRNAs, miRNAs and mRNAs detected by RNA-seq, respectively. The expression values from − 2 to 2 were depicted in line with the color scale representing the intensity increased from green to red. Three osteogenic induced groups and three control groups were included, each column represented one sample and each row indicated one transcript. Bioinformatics software (http://www.bioinformatics.com.cn/) was used to create the maps in Fig. 3B ~ E

Based on the sequencing data, 1524 lncRNAs were differentially expressed, of which 812 up-regulated and 712 down-regulated in osteogenic induced groups compared with those in control groups. In addition, 30 up-regulated and 61 down-regulated miRNAs, a total of 91 miRNAs were also showed differential expression. Meanwhile, 2453 differentially expressed mRNAs were explored, including 1272 up-regulated and 1181 down-regulated expression (Fig. 3A). The volcano map manifested differentially expressed lncRNAs, miRNAs and mRNAs with statistical significance (*P* < 0.05) and |fold change|>2 (Fig. 3B). The heat map also showed the differences in lncRNAs (Fig. 3C), miRNAs (Fig. 3D) and mRNAs expression (Fig. 3E) between 14-day osteogenic-induced and control groups. The apparent difference of transcript levels between these two groups indicated the crucial roles of lncRNAs, miRNAs and mRNAs during the rBMMSCs osteogenic process.

### Selection of lncRNAs and the prediction of lncRNA-miRNA interactions

**Fig. 4:**
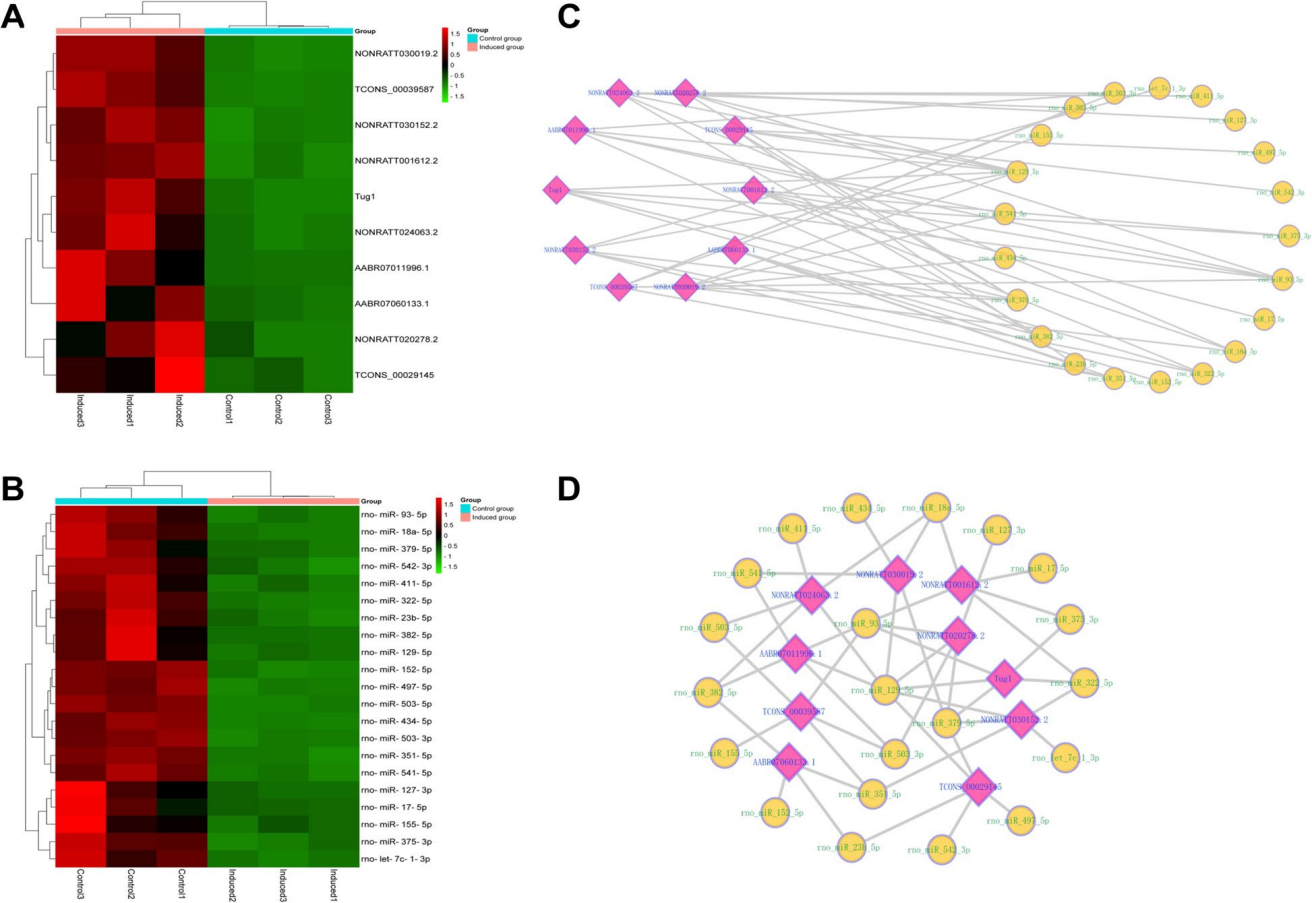
Expression profiles of 10 lncRNAs and 21 miRNAs and construction of their potential networks. **A**, The heat map of 10 lncRNAs. **B**, The heat map of 21 miRNAs. These two heat maps were constructed based on expression values of 10 expressed lncRNAs and 21 miRNAs, respectively. The expression values from − 1.5 to 1.5 were depicted in line with the color scale representing the intensity increased from green to red. Three osteogenic induced groups and three control groups were included, each column represented one sample and each row indicated one transcript. **C**, Construction of potential lncRNA–miRNA networks based on 10 lncRNAs and 21 miRNAs by group type attributes layout exporting. **D**, Construction of potential lncRNA–miRNA networks based on 10 lncRNAs and 21 miRNAs by interaction attributes layout exporting. Bioinformatics software (http://www.bioinformatics.com.cn/) was used to create the maps in Fig. 4A ~ B. Cytoscape 3.9.0 software (https://cytoscape.org/) was used to create the maps in Fig. 4C ~ D

In order to verify the key sequencing data in the subsequent experiment, we initially selected 10 lncRNAs, including NONRATT001612.2, AABR07060133.1, NONRATT030019.2, TCONS_00039587, NONRATT030152.2, Tug1, AABR07011996.1, NONRATT024063.2, NONRATT020278.2 and TCONS_00029145, from 812 up-regulated lncRNAs based on fold change, raw intensity and relevant literature evidence [26]. The results displayed by the heat map (Fig. 4A). The original data can be seen in Supplementary material 1. Since lncRNAs can function as miRNA “sponges” to mediate gene expression, we screened 34 miRNAs with the criteria of *P* < 0.05, |log_2_fold change|>1 and self-expression level > 1, from 61 down-regulated miRNAs in sequencing data. The interaction relationships between these 10 lncRNAs and 34 miRNAs were predicted by RNA22 v2 microRNA target detection software, and the top 5 miRNAs were chosen for each lncRNA according to the smaller *P* value (Table 2). What needs to be mentioned is lncRNA AABR07060133.1, because it has only four predicted miRNAs that could be targeted. As results exported by heatmap, a total of 21 miRNAs were screened out that may be combined with those 10 lncRNAs (Fig. 4B). The original data can be seen in Supplementary material 1. Finally, we obtained 49 lncRNA-miRNA pairs based on the sequencing results and bioinformatic predictions, and the potential lncRNA–miRNA interactive networks were constructed by Cytoscape 3.9.0 software in two manifestations (Fig. 4C, D).

**Table 2:** The selected 10 upregulated lncRNAs and their putative downregulated miRNAs during 14-day osteogenic differentiation of rBMMSCs

LncRNA	MRE1	*P* value	MRE2	*P* value	MRE3	*P* value	MRE4	*P* value	MRE5	*P* value
NONRATT001612.2	miR-375-3p	0.009	miR-93-5p	0.102	miR-17-5p	0.102	miR-18a-5p	0.102	miR-322-5p	0.112
AABR07060133.1	miR-152-5p	0.019	miR-351-5p	0.104	miR-23b-5p	0.207	miR-382-5p	0.311	-	-
NONRATT030019.2	miR-379-5p	0.005	miR-434-5p	0.007	miR-541-5p	0.007	miR-18a-5p	0.007	miR-129-5p	0.025
TCONS-00039587	miR-155-5p	0.023	miR-503-5p	0.023	miR-503-3p	0.023	miR-93-5p	0.039	miR-351-5p	0.039
NONRATT030152.2	miR-129-5p	0.002	miR-379-5p	0.011	miR-351-5p	0.011	miR-322-5p	0.011	let-7c-1-3p	0.011
Tug1	miR-375-3p	0.003	miR-379-5p	0.005	miR-129-5p	0.018	miR-322-5p	0.036	miR-93-5p	0.136
AABR07011996.1	miR-93-5p	0.001	miR-541-5p	0.030	miR-503-3p	0.055	miR-382-5p	0.056	miR-129-5p	0.058
NONRATT024063.2	miR-382-5p	0.024	miR-503-5p	0.024	miR-411-5p	0.024	miR-18a-5p	0.035	miR-129-5p	0.055
NONRATT020278.2	miR-93-5p	0.015	miR-379-5p	0.088	miR-129-5p	0.099	miR-503-3p	0.166	miR-127-3p	0.166
TCONS-00029145	miR-129-5p	0.001	miR-23b-5p	0.011	miR-497-5p	0.011	miR-542-3p	0.011	miR-379-5p	0.020

### Prediction of miRNA-mRNA interactions

**Fig. 5:**
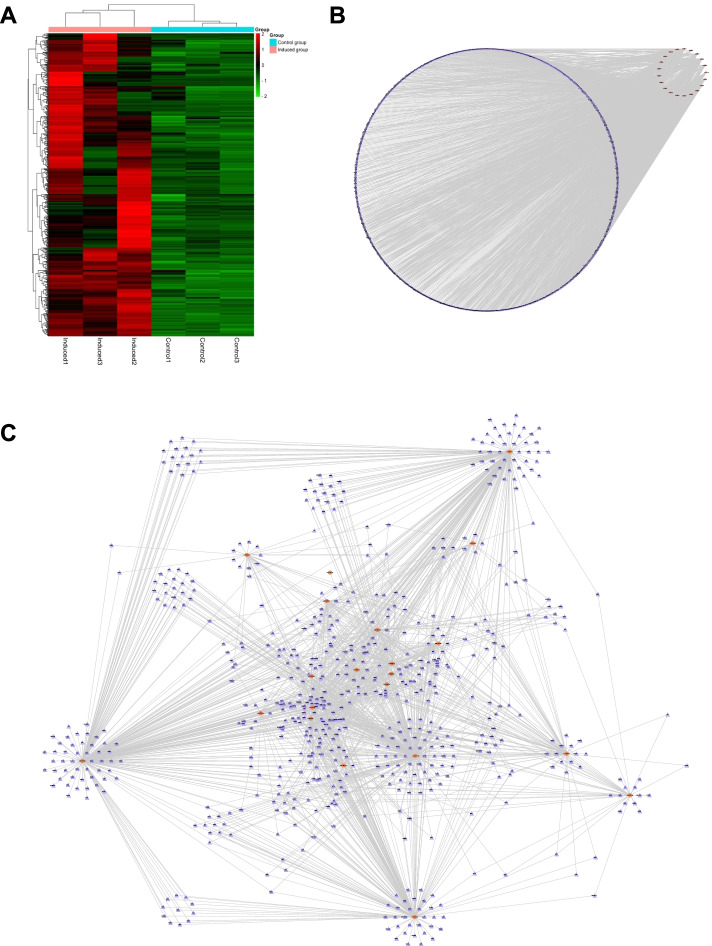
Expression profile of 650 mRNAs and potential miRNA-mRNA networks construction. **A**, The heat map of 650 mRNAs. This heat map was constructed based on expression values of 650 miRNAs. The expression values from − 2 to 2 were depicted in line with the color scale representing the intensity increased from green to red. Three osteogenic induced groups and three control groups were included, each column represented one sample and each row indicated one transcript. **B**, Construction of potential miRNA–mRNA networks based on 21 miRNAs and 650 mRNAs by group type attributes layout exporting. **C**, Construction of potential miRNA–mRNA networks based on 21 miRNAs and 650 mRNAs by interaction attributes layout exporting. Bioinformatics software (http://www.bioinformatics.com.cn/) was used to create the maps in Fig. 5A. Cytoscape 3.9.0 software (https://cytoscape.org/) was used to create the maps in Fig. 5B ~ C

To determine the further molecular mechanisms, potential mRNAs binding with those 21 miRNAs were investigated. The miRNA-mRNA binding data were obtained from TargetScan 8.0 and miRDB databases, and 650 mRNAs overlapped with the up-regulated mRNAs of sequencing data by Venn analysis, their differential expression was presented by heatmap (Fig. 5A). The original data can be seen in Supplementary material 1. Then, 1515 miRNA–mRNA pairs interactive networks in two manifestations were constructed by aforementioned 21 miRNAs and 650 mRNAs (Fig. 5B, C).

### Functional prediction of interactive mRNAs

**Fig. 6:**
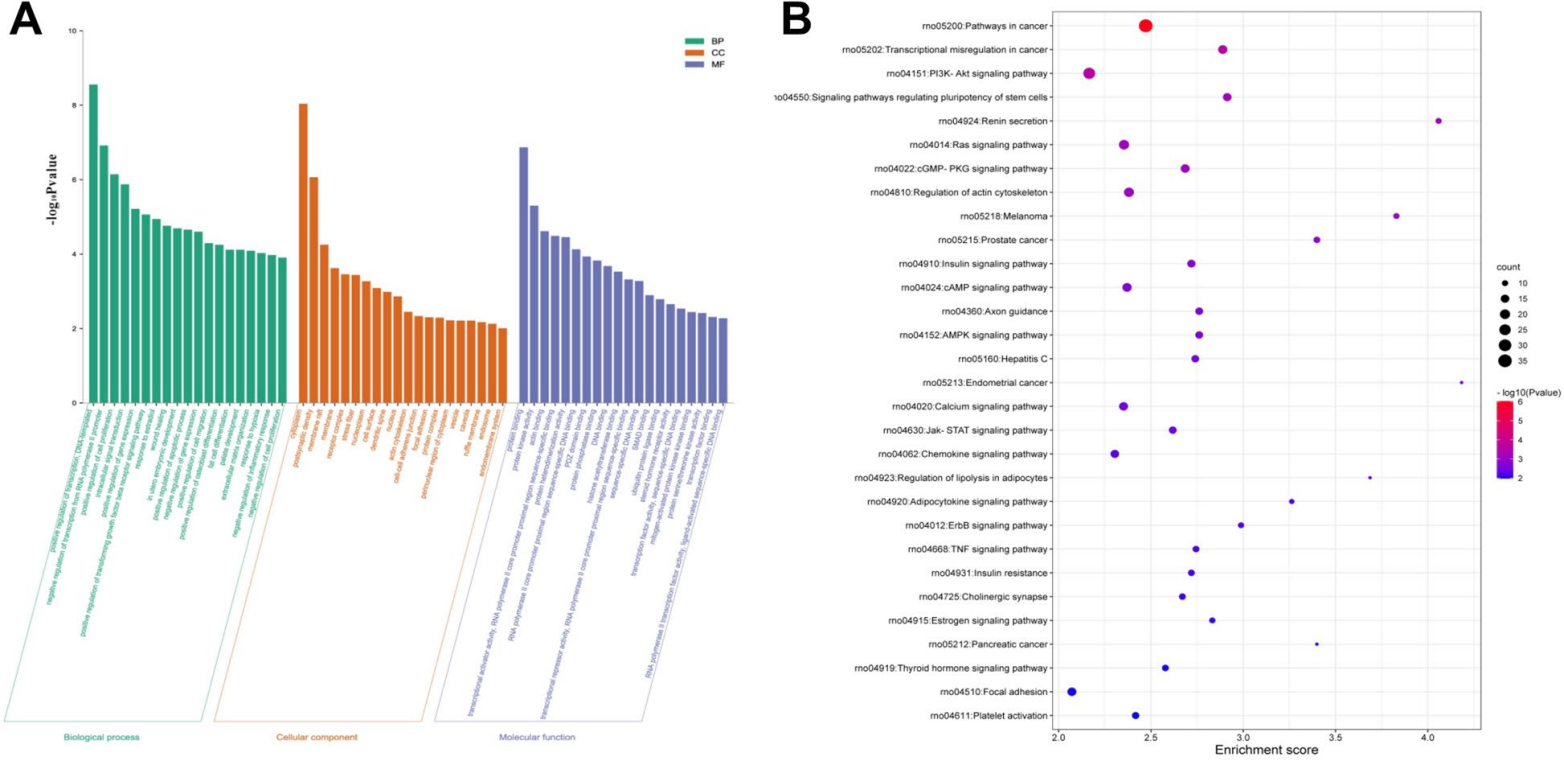
Functional analysis of 650 mRNAs. **A**, The top 20 enrichment GO terms for each category including biological process, cellular component and molecular function ranked by -log10 (*P* value) through GO analysis. **B**, The top 30 enrichment KEGG pathways ranked by enrichment score. Bioinformatics software (http://www.bioinformatics.com.cn/) was used to create the maps in Fig. 6A ~ B. KEGG software (https://www.genome.jp/kegg/) was used to apply the source information

The interactive 650 mRNAs were further selected to perform GO enrichment and KEGG pathways enrichment, evaluating the role of mRNAs in biological processes, cellular components, molecular functions, as well as in pathways. In general, 455 GO terms were analyzed, including 320 terms enrichment in biological process, 80 terms enrichment in molecular function and 55 terms enrichment in cellular component. The significant enrichment histogram showed that a total of 60 GO items were added by the top 20 items for each category ranked by enrichment score (Fig. 6A). The most significantly enriched GO term in the cellular component was “cytoplasm”, while “protein binding” was the most significantly enriched GO term in the molecular function. Furthermore, biological processes such as “cell proliferation”, “wound healing”, “cell migration”, “osteoblast differentiation”, “extracellular matrix organization” and “response to hypoxia” have been also enriched. The original data can be seen in Supplementary material 2.

By KEGG pathways analysis, 68 significant pathways were detected and ranked by enrichment score with the top 30 pathways listed (Fig. 6B). Among them, the pathway category “PI3K-Akt signaling pathway”, “Signaling pathways regulating pluripotency of stem cells”, “cGMP-PKG signaling pathway”, “Axon guidance”, and “Calcium signaling pathway” have previously been reported during osteogenic process [27–31]. The original data can be seen in Supplementary material 2.

### Expression validation and lncRNA-miRNA-mRNA co-expression networks construction

**Fig. 7:**
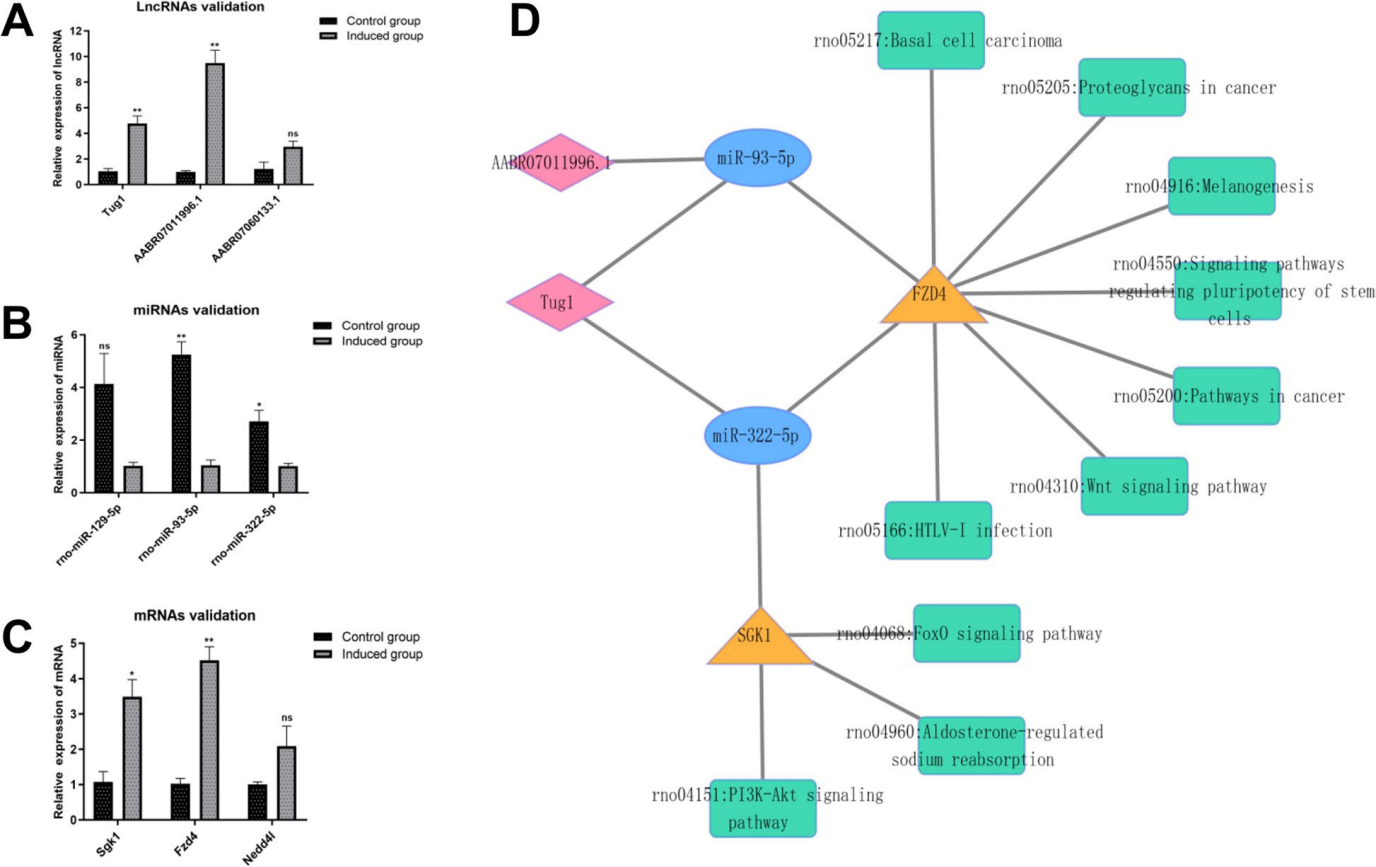
Expression validation and lncRNA-miRNA-mRNA co-expression networks construction. **A**, qRT-PCR validation of 3 lncRNAs. **B**, qRT-PCR validation of 3 miRNAs. **C**, qRT-PCR validation of 3 mRNAs. **D**, Construction of potential interactive networks with pathways enrichment based on 2 lncRNAs, 2 miRNAs, and 2 mRNAs. Independent t test was used in qRT-PCR validation. *P* < 0.05 considered significant difference, and ns indicated that the difference is not statistically significant. Cytoscape 3.9.0 software (https://cytoscape.org/) was used to create the maps in Fig. 7B

According to the prediction of lncRNA-miRNA-mRNA networks extended by the selected 10 lncRNAs mentioned above, we eventually selected 3 lncRNAs (Tug1, AABR07060133.1 and AABR07011996.1) for qRT-PCR validation, and the results showed that their expression were up-regulated (Fig. 7A). lncRNAs Tug1 and AABR07011996.1 showed statistically significant differences in expression between the two groups (*P* < 0.05), while lncRNA AABR07060133.1 showed no significant difference (*P* > 0.05). Meanwhile, we picked 3 miRNAs out (rno-miR-129-5p, rno-miR-93-5p and rno-miR-322-5p), and verified their down-regulated expression (Fig. 7B). The expression differences of two miRNAs (rno-miR-93-5p and rno-miR-322-5p) were statistically significant (*P* < 0.05). However, there was no significant difference in the expression of rno-miR-129-5p (*P* > 0.05). Moreover, 3 mRNAs (Nedd4l, Sgk1and Fzd4) were picked to verify based on the prediction of combining with afore-selected miRNAs. The results showed that the other 2 mRNAs (*P* < 0.05) except Nedd4l displayed up-regulated expression with significant difference (Fig. 7C). In consequence, 2 lncRNAs and 2 mRNAs showed differential upregulation, while 2 miRNAs showed differential downregulation in osteogenic-induced group when compared with control group (*P* < 0.05). Owing to the verified data, 2 lncRNAs, 2 miRNAs and 2 mRNAs constructed 4 links of potential co-expression networks. In addition, the possible enrichment pathways were identified (Fig. 7D).

## Discussion

BMMSCs are regarded as significant seed cells in the field of bone tissue engineering owing to their multi-differentiation potential. They are usually genetically modified to participate in the mediation of bone formation. Over the last decade, accumulating evidences have shown that lncRNAs play diversity roles on cell proliferation, migration, and osteoblasts differentiation [32, 33]. Taking the diverse regulatory modes and biological functions of lncRNAs on bone homeostasis into account, it has become the research hotspot. As is well-known, the expression of lncRNAs with cell specificity, tissue specificity and developmental stage specificity, and can affect different biological processes [34]. In terms of the osteogenic developmental stage, commonly accepted models include early proliferative stage (0 ~ 4 days), matrix maturation stage (4 ~ 14 days), and mineralization stage (14 ~ 21 days), that indicate the transition of mesenchymal stem cells to pro-osteoblasts, early osteoblasts, and mature osteoblasts [35]. Consequently, 14-day osteogenic induction may be related to the late stage of matrix maturation and the early stage of mineralization, and this stage may be associated with the transition of mesenchymal stem cells to early osteoblasts and mature osteoblasts. Thus, the differentially expressed lncRNAs in 14-day osteogenic developmental stage are worthy of our study.

Actually, except for lncRNAs, miRNAs also showed the significant roles on osteogenesis, because they can act as “sponge” combining with lncRNAs to relieve the suppression on target mRNAs expression, which involved in multiple mechanisms. For example, lncRNA HHAS1 enhanced Runx2 level by downregulating miR-204-5p expression to facilitate osteogenic differentiation [36], while lncRNA CCAT1 was found to prevent smurf2 degradation by bonding with miR-34a-5p, suppressing differentiation of osteoblasts [37]. The above examples showed the importance of miRNAs in regulating osteoblast differentiation by lncRNAs. Understanding the regulation mechanisms that control the differentiation of rBMMSCs into osteoblasts, including lncRNAs, miRNAs and mRNAs, is critical for bone regeneration.

Considering that comprehensive analysis of differentially expression of lncRNAs, miRNAs and mRNAs in rBMMSCs for 14-day osteogenic induction have not yet been reported. In this study, we explored the expression profiles of lncRNAs, miRNAs and mRNAs in rBMMSCs under 14-day osteogenic induction and non-osteogenic culturation using high-throughput RNA sequencing, which allows us to detect all RNA transcripts. Unlike microarray, a method which is limited by pre-designed probes. Finally, we identified 8634 lncRNAs through four kinds of coding potential analysis methods intersection, and classified four types of lncRNAs to facilitate subsequent functional prediction. The most of identified lncRNAs showed lower exon number and shorter than 2000nt transcript length, which is consistent with the results from other literatures [4, 38].

Additionally, differentially expressed lncRNAs, miRNAs and mRNAs were also identified for predicting ceRNA mechanisms and potential functions. Due to such a large number of differentially expressed data detected, we initially selected 10 lncRNAs and predicted 21 miRNAs and 650 targeted mRNAs in order to narrow the scope of subsequent experimental verification. A total of 49 lncRNA-miRNA pairs and 1515 miRNA–mRNA pairs interactive networks were constructed, which were used as the basis for further selection of lncRNAs, miRNAs and mRNAs, this may facilitate to improve the possibility and pluripotency on subsequent validation data.

To clarify the potential functions, GO analysis was used to further annotate the biological processes, cellular components and molecular functions of those differentially expressed 650 mRNAs. Consistent with a recent report [38], BP terms that include “positive regulation of transcription” and “negative regulation of transcription” were highly enriched, suggesting that these mRNAs may directly target transcriptional activators or repressors to regulate their downstream targets. Besides, the BP terms including “cell proliferation” with 40 significant genes, “intracellular signal transduction” with 33 significant genes, “wound healing” with 16 significant genes, “cell migration” with 20 significant genes, “osteoblast differentiation” with 11 significant genes, “extracellular matrix organization” with 13 significant genes and “response to hypoxia” with 23 significant genes were reported as important activities in bone formation [39–42], and their significant genes were worthy to be investigated. In terms of CC terms, the most enriched was cytoplasm. Extracellular matrix (ECM) was also mentioned. It is always regarded as the growth factors reservoir by establishing stable gradients for mesenchymal stem cells osteogenic differentiation [43]. As for MF terms describing molecular biological activity, it mainly covers catalytic activity, transport activity and binding activity. In this study, except for protein binding was found to be the most important enrichment, DNA binding, transcription factor binding, and SMAD binding were also detected in the top 20 terms, indicating that differentially expressed lncRNAs and their potential target genes may have diverse functions in the process of osteogenesis.

Furthermore, KEGG analysis was able to provide hints for the pathway enrichments, it can find out which cellular pathway changes the differential transcripts may be related to. Here, many crucial signaling pathways associated with osteogenic differentiation were enriched. Particular speaking, “PI3K-Akt signaling pathway”, the most important osteogenesis-related enrichment among the top 10 pathways, since current researches [44, 45] proved that the activation of the PI3K/AKT signaling pathway can promote osteogenesis, indicating the profound significance of this pathway for bone regeneration, which also paves the way for future studies. Other enrichment pathways, such as “Signaling pathways regulating pluripotency of stem cells” [44], “cGMP-PKG signaling pathway” [29], “Axon guidance” [28], and “Calcium signaling pathway” [42] have also been reported to be involved in the process of osteogenic differentiation, providing a preliminary basis for future research.

According to the afore-constructed networks of potential interactions, 3 up-regulated lncRNAs (Tug1, AABR07060133.1, AABR07011996.1), 3 down-regulated miRNAs (rno-miR-129-5p, rno-miR-93-5p, rno-miR-322-5p) and 3 up-regulated mRNAs (Nedd4l, Sgk1, Fzd4) were selected to examine their expression levels using qRT-PCR. It has to be said that the selection of rno-miR-129-5p, rno-miR-93-5p and rno-miR-322-5p is not only due to the multiple degrees of connection with the afore-selected lncRNAs, but also reported that they may negatively regulate bone formation [46–48]. Additionally, they were also preliminary proved in our results on 14-day osteogenic induction of rBMMSCs. Fortunately, the most validation results of qRT-PCR were consistent with the sequencing data which proved the reliability of the sequencing data. Meanwhile, the corresponding co-expression networks constructed by 2 lncRNAs, 2 miRNAs and 2 mRNAs indicated that complicated regulatory relationships between lncRNAs, miRNAs and mRNAs, where one lncRNA could regulate multiple miRNAs and one miRNA could regulate multiple mRNAs, while one mRNA could be regulated by multiple miRNAs and one miRNA could be regulated by multiple lncRNAs. When lncRNA and mRNA had the same MRE, the regulatory mechanism of ceRNA will be revealed that lncRNAs can competitively adsorb miRNAs like sponges, reduce the inhibitory effect of miRNAs on target mRNAs, and increase the expression level of target mRNAs. The complex regulatory relationship between them is attributed to the existence of ceRNA regulatory mechanism, which is also a key content of our subsequent research. Moreover, the enrichment of “PI3K-Akt signaling pathway”, “Signaling pathways regulating pluripotency of stem cells” and “Wnt signaling pathway” were observed based on the validations, further demonstrating the possibility of those pathways regulating osteogenesis process. However, these results need to be verified in the future.

Recently, the importance of our validated mRNAs for osteogenic differentiation process has been proved to an extent. The hyperglycemia of diabetes can stimulate Sgk1 expression, augmenting store-operated Ca^2+^-entry and osteogenic signaling in hAoSMCs [49]. Inhibition of Sgk1 can repress osteoclastogenesis in vitro and prevent bone loss in vivo [50]. Voelkl, J., et al., [51] identified Sgk1 as a key regulator of vascular calcification, its increase can promote vascular calcification via NF-κB activation and its decrease can reduce the burden of vascular calcification in chronic kidney disease (CKD). Kushwaha et al. [52] indicated that Fzd4 is required for normal bone development and mineralization. Further research found that Fzd4 not only mediate the mechanical stretch-induced osteogenic differentiation of BMMSCs [53], but also promote proliferation and osteogenic differentiation of hBMMSCs by Wnt/β-catenin pathway [54, 55]. Mechanistically, Fzd4 was identified as the potential target of miR-1292, promoting hADSCs osteogenesis through the Wnt/β-catenin signaling pathway [56]. Also, it was the potential target of miR-129-5p [57], miR-144-3p [58] and miR-139-5p [59], regulating BMMSCs osteoblast differentiation.

Here are some limitations in this study. On one hand, lacking of more biological basis of aforementioned networks presently. Some additional results will be provided to support our findings related to osteogenic differentiation in the future. On the other hand, GO and KEGG can only indicate functional difference, and this is still at gene expression level. Further understanding of the functional role and regulatory mechanism of target lncRNA in the process of rBMMSCs osteogenic differentiation will be the main content of our subsequent research, which will be reflected in our later research results. Consequently, this study is only a preliminary study based on RNA-seq technology and bioinformatic analysis, aiming to find new lncRNAs, miRNAs and mRNAs potentially being related to 14-day osteogenic differentiation, screen some novel lncRNA-miRNA-mRNA networks to reduce the scope of gene verification and improve verification effect, scientifically screen the target lncRNA and provide new reference information for further study on the function and regulation mechanism of target lncRNA.

## Conclusions

In this study, we identified the expression profiles of lncRNAs, miRNAs and mRNAs during 14-day osteogenic differentiation of rBMMSCs, and calculated their differential expressions. According to the RNA-seq results and bioinformatic predictions, we constructed the potential lncRNA-miRNA and miRNA-mRNA networks through choosing 10 up-regulated lncRNAs as clues to narrow the selection scape of subsequent validation. Their potential functions were predicted by GO and KEGG enrichment. At last, 3 lncRNAs, 3 miRNAs and 3 mRNAs were selected to verify. Based on the verification, their corresponding pathways were enriched. This may better understand their possible regulatory mechanisms, providing new reference for our further studies.

In a word, this is the first report on the expression profiles of lncRNAs, miRNAs and mRNAs in rBMMSCs under 14-day osteogenic induction using high-throughput RNA sequencing, which can provide scientific methodological basis and reliable data support for subsequent deeper researches.

In addition, lncRNA-miRNA-mRNA interactive networks may provide more information on novel targets and mechanisms for clinical treatments on bone regeneration.

## Supplementary Information

**Additional file 1.**

**Additional file 2.**

## Data Availability

(ADM) All data generated or analyzed during this study are included in this article.

## Abbreviations

BMMSCs: Bone marrow mesenchymal stem cells
BP: Biological process
CC: Cellular component
ceRNA: Competing endogenous RNA
ECM: Extracellular matrix
GO: Gene ontology
KEGG: Kyoto encyclopedia of genes and genomes
lncRNA: Long non-coding RNA
miRNA: Micro RNA
mRNA: Messenger RNA
MRE: miRNA response element
MF: Molecular function
qRT-PCR: Real-time quantitative reverse transcription-polymerase chain reaction
RNA-seq: High-throughput RNA sequencing
rno: Rattus norvegicus
rBMMSCs: rat Bone marrow mesenchymal stem cells

## References

[1] Arthur A, Gronthos S. Clinical Application of Bone Marrow Mesenchymal Stem/Stromal Cells to Repair Skeletal Tissue. Int J Mol Sci. 2020;21(24):9759. 10.3390/ijms21249759 PMC7767389 33371306

[2] Li L (2021). LINC00370 modulates miR-222-3p-RGS4 axis to protect against osteoporosis progression. Arch Gerontol Geriatr.

[3] Sun J (2021). Long noncoding RNA AC092155 facilitates osteogenic differentiation of adipose-derived stem cells through the miR-143-3p/STMN1 axis. J Gene Med.

[4] Liu Z (2020). Differential expression of lncRNA/miRNA/mRNA and their related functional networks during the osteogenic/odontogenic differentiation of dental pulp stem cells. J Cell Physiol.

[5] Sun X (2019). Potential functions of long noncoding RNAs in the osteogenic differentiation of human bone marrow mesenchymal stem cells. Mol Med Rep.

[6] Li Z, Guo X, Wu S (2020). Epigenetic silencing of KLF2 by long non-coding RNA SNHG1 inhibits periodontal ligament stem cell osteogenesis differentiation. Stem Cell Res Ther.

[7] Ru J (2020). Hydrostatic pressure induces osteogenic differentiation of adipose-derived mesenchymal stem cells through increasing lncRNA-PAGBC. Aging (Albany NY).

[8] Cao L (2020). Linc02349 promotes osteogenesis of human umbilical cord-derived stem cells by acting as a competing endogenous RNA for miR-25-3p and miR-33b-5p. Cell Prolif.

[9] Chen Z (2020). lncRNA HOTAIRM1 promotes osteogenesis of hDFSCs by epigenetically regulating HOXA2 via DNMT1 in vitro. J Cell Physiol.

[10] Kong L, et al. CPC: assess the protein-coding potential of transcripts using sequence features and support vector machine. Nucleic Acids Res. 2007;35(Web Server issue):W345-9. 10.1093/nar/gkm391 PMC1933232 17631615

[11] Sun L (2013). Utilizing sequence intrinsic composition to classify protein-coding and long non-coding transcripts. Nucleic Acids Res.

[12] El-Gebali S (2019). The Pfam protein families database in 2019. Nucleic Acids Res.

[13] Li A, Zhang J, Zhou Z. PLEK: a tool for predicting long non-coding RNAs and messenger RNAs based on an improved k-mer scheme. 2014. p. 311. 10.1186/1471-2105-15-311 PMC4177586 25239089

[14] Trapnell C (2010). Transcript assembly and quantification by RNA-Seq reveals unannotated transcripts and isoform switching during cell differentiation. Nat Biotechnol.

[15] Huber W, et al. Orchestrating high-throughput genomic analysis with Bioconductor. 2015; p. 115–121. 10.1038/nmeth.3252 PMC4509590 25633503

[16] Miranda KC (2006). A pattern-based method for the identification of MicroRNA binding sites and their corresponding heteroduplexes. Cell.

[17] Agarwal V, et al. Predicting effective microRNA target sites in mammalian mRNAs. Elife. 2015;12(4):e05005. 10.7554/eLife.05005 PMC4532895 26267216

[18] McGeary SE, et al. The biochemical basis of microRNA targeting efficacy. Science. 2019;366(6472):eaav1741. 10.1126/science.aav1741 PMC7051167 31806698

[19] Liu W, Wang X (2019). Prediction of functional microRNA targets by integrative modeling of microRNA binding and target expression data. Genome Biol.

[20] Chen Y, Wang X (2020). miRDB: an online database for prediction of functional microRNA targets. Nucleic Acids Res.

[21] Ashburner M (2000). Gene ontology: tool for the unification of biology. The Gene Ontology Consortium. Nat Genet.

[22] The Gene Ontology (2021). resource: enriching a GOld mine. Nucleic Acids Res.

[23] Kanehisa M, Goto S (2000). KEGG: kyoto encyclopedia of genes and genomes. Nucleic Acids Res.

[24] Kanehisa M (2017). KEGG: new perspectives on genomes, pathways, diseases and drugs. Nucleic Acids Res.

[25] Arocho A (2006). Validation of the 2-DeltaDeltaCt calculation as an alternate method of data analysis for quantitative PCR of BCR-ABL P210 transcripts. Diagn Mol Pathol.

[26] Du H (2021). LncRNA TUG1 silencing enhances proliferation and migration of ox-LDL-treated human umbilical vein endothelial cells and promotes atherosclerotic vascular injury repairing via the Runx2/ANPEP axis. Int J Cardiol.

[27] Dong K (2021). The extract of concentrated growth factor enhances osteogenic activity of osteoblast through PI3K/AKT pathway and promotes bone regeneration in vivo. Int J Implant Dent.

[28] Wan QQ (2021). Crosstalk between Bone and Nerves within Bone. Adv Sci (Weinh).

[29] Kim SM (2021). The NO-cGMP-PKG pathway in skeletal remodeling. Ann N Y Acad Sci.

[30] Zhao H (2021). Hypoxia Enhanced Bone Regeneration Through the HIF-1alpha/beta-Catenin Pathway in Femoral Head Osteonecrosis. Am J Med Sci.

[31] Kim HJ (2021). Flunarizine inhibits osteoclastogenesis by regulating calcium signaling and promotes osteogenesis. J Cell Physiol.

[32] Niu S, Xiang F, Jia H (2021). Downregulation of lncRNA XIST promotes proliferation and differentiation, limits apoptosis of osteoblasts through regulating miR-203-3p/ZFPM2 axis. Connect Tissue Res.

[33] Zheng J (2021). lncRNASNHG7003 inhibits the proliferation, migration and invasion of vascular smooth muscle cells by targeting the miR13065p/SIRT7 signaling pathway. Int J Mol Med.

[34] Quinn JJ, Chang HY (2016). Unique features of long non-coding RNA biogenesis and function. Nat Rev Genet.

[35] Long T (2018). Differential Expression Profiles of Circular RNAs During Osteogenic Differentiation of Mouse Adipose-Derived Stromal Cells. Calcif Tissue Int.

[36] Ye G (2021). IRF2-mediated upregulation of lncRNA HHAS1 facilitates the osteogenic differentiation of bone marrow-derived mesenchymal stem cells by acting as a competing endogenous RNA. Clin Transl Med.

[37] Hu F (2021). Silencing long noncoding RNA colon cancer-associated transcript-1 upregulates microRNA-34a-5p to promote proliferation and differentiation of osteoblasts in osteoporosis. Cancer Gene Ther.

[38] Wang H (2021). Analysis of lncRNAs-miRNAs-mRNAs networks in periodontal ligament stem cells under mechanical force. Oral Dis.

[39] Chen C (2021). HIF/Ca(^2+^)/NO/ROS is critical in roxadustat treating bone fracture by stimulating the proliferation and migration of BMSCs. Life Sci.

[40] Torres P (2021). Histatin-1 is a novel osteogenic factor that promotes bone cell adhesion, migration, and differentiation. J Tissue Eng Regen Med.

[41] Chen K (2021). Osteoblast-derived EGFL6 couples angiogenesis to osteogenesis during bone repair. Theranostics.

[42] Wu L (2020). Intracellular Ca(^2+^) signaling mediates IGF-1-induced osteogenic differentiation in bone marrow mesenchymal stem cells. Biochem Biophys Res Commun.

[43] Miar S, et al. Regeneration enhanced in critical-sized bone defects using bone-specific extracellular matrix protein. 2021;538–547. 10.1002/jbm.b.34722 PMC8740960 32915522

[44] Xu J (2021). Low-dose IL-34 has no effect on osteoclastogenesis but promotes osteogenesis of hBMSCs partly via activation of the PI3K/AKT and ERK signaling pathways. Stem Cell Res Ther.

[45] Sun Z (2021). MiR-126 affects femoral fracture healing in rats through PI3K/AKT signaling pathway. Panminerva Med.

[46] Yin C (2020). miR-129-5p Inhibits Bone Formation Through TCF4. Front Cell Dev Biol.

[47] Zhang Y (2017). Increased microRNA-93-5p inhibits osteogenic differentiation by targeting bone morphogenetic protein-2. PLoS One.

[48] Zeng Y, et al. Sox9-Increased miR-322-5p Facilitates BMP2-Induced Chondrogenic Differentiation by Targeting Smad7 in Mesenchymal Stem Cells. Stem Cells Int. 2021; 2021:9778207. 10.1155/2021/9778207 PMC8589527 34777504

[49] Ma K, et al. Stimulation of ORAI1 expression, store-operated Ca entry, and osteogenic signaling by high glucose exposure of human aortic smooth muscle cells. 2020;1093–1102. 10.1007/s00424-020-02405-1 32556706

[50] Zhang Z, et al. Serum- and Glucocorticoid-inducible Kinase 1 is Essential for Osteoclastogenesis and Promotes Breast Cancer Bone Metastasis. 2020;650–660. 10.1158/1535-7163.MCT-18-0783 31694887

[51] Voelkl J, et al. SGK1 induces vascular smooth muscle cell calcification through NF-κB signaling. 2018;3024–3040. 10.1172/JCI96477 PMC6025998 29889103

[52] Kushwaha P, et al. Frizzled-4 is required for normal bone acquisition despite compensation by Frizzled-8. 2020;6673–6683. 10.1002/jcp.29563 PMC7382978 31985040

[53] Gu Q, et al. Wnt5a/FZD4 Mediates the Mechanical Stretch-Induced Osteogenic Differentiation of Bone Mesenchymal Stem Cells. 2018;215–226. 10.1159/000491721 30007964

[54] Zhang Z, et al. Circ_FBLN1 promotes the proliferation and osteogenic differentiation of human bone marrow-derived mesenchymal stem cells by regulating let-7i-5p/FZD4 axis and Wnt/β-catenin pathway. 2021;561–572. 10.1007/s10863-021-09917-0 34424449

[55] Gao H, et al. LINC01119 negatively regulates osteogenic differentiation of mesenchymal stem cells via the Wnt pathway by targeting FZD4. 2022;43. 10.1186/s13287-022-02726-1 PMC8800246 35093173

[56] Fan J, et al. MiR-1292 Targets FZD4 to Regulate Senescence and Osteogenic Differentiation of Stem Cells in TE/SJ/Mesenchymal Tissue System via the Wnt/β-catenin Pathway. 2018;1103–1121. 10.14336/AD.2018.1110 PMC6284756 30574422

[57] Wang J, et al. miR-129-5p in exosomes inhibits diabetes-associated osteogenesis in the jaw via targeting FZD4. 2021;87–93. 10.1016/j.bbrc.2021.05.072 34119828

[58] Sun Z, et al. MiR-144-3p Inhibits BMSC Proliferation and Osteogenic Differentiation Targeting FZD4 in Steroid-Associated Osteonecrosis. 2019;4806–4812. 10.2174/1381612825666190930094019 31566128

[59] Long H, et al. miR-139-5p Represses BMSC Osteogenesis via Targeting Wnt/β-Catenin Signaling Pathway. 2017;715–724. 10.1089/dna.2017.3657 28622009

